# Hepatopulmonary syndrome in patients with chronic liver disease: role of pulse oximetry

**DOI:** 10.1186/1471-230X-6-15

**Published:** 2006-04-25

**Authors:** Peter Deibert, Hans-Peter Allgaier, Stefanie Loesch, Claudia Müller, Manfred Olschewski, Hinrich Hamm, Klaus-Peter Maier, Hubert Erich Blum

**Affiliations:** 1 Department of Medicine VII, University Freiburg, Germany; 2 Department of Medicine II, University Freiburg, , Germany; 3 Department of Medical Biometry, University Freiburg, , Germany; 4 Helios Hospital, Department of Internal Medicine, Titisee-Neustadt, Germany; 5 Liver Center, City Hospital Esslingen, Germany; 6 Asklepios Klinik, Dept. of Internal Medicine and Lung Clinic, Westerland, Germany

## Abstract

**Background:**

Hepatopulmonary syndrome (HPS) is a rare complication of liver diseases of different etiologies and may indicate a poor prognosis. Therefore, a simple non-invasive screening method to detect HPS would be highly desirable. In this study pulse oximetry was evaluated to identify patients with HPS.

**Methods:**

In 316 consecutive patients with liver cirrhosis (n = 245), chronic hepatitis (n = 69) or non-cirrhotic portal hypertension (n = 2) arterial oxygen saturation (SaO_2_) was determined using a pulse oximeter. In patients with SaO_2 _≤92% in supine position and/or a decrease of ≥4% after change from supine to upright position further diagnostic procedures were performed, including contrast-enhanced echocardiography and perfusion lung scan.

**Results:**

Seventeen patients (5.4%) had a pathological SaO_2_. Four patients (1.3%) had HPS. HPS patients had a significant lower mean SaO_2 _in supine (89.7%, SD 5.4 *vs*. 96.0%, SD 2.3; p = 0.003) and upright position (84.3%, SD 5.0 *vs*. 96.0%, SD 2.4; p = 0.001) and had a lower mean PaO_2 _(56.2 mm Hg, SD 15.2 *vs*. 71.2 mm Hg, SD 20.2; p = 0.02) as compared to patients without HPS. The mean ΔSaO_2 _(difference between supine and upright position) was 5.50 (SD 7) in HPS patients compared to non-HPS patients who showed no change (p = 0.001). There was a strong correlation between shunt volume and the SaO_2 _values (R = -0.94).

**Conclusion:**

Arterial SaO_2 _determination in supine and upright position is a useful non-invasive screening test for HPS and correlates well with the intrapulmonary shunt volume.

## Background

In 1884 Flückiger first described a women with liver cirrhosis, cyanosis, and digital clubbing [1]. The term 'hepatopulmonary syndrome', the triad of liver disease, an increased alveolar-arterial gradient while breathing room air, and evidence of intrapulmonary vascular dilatations, was coined in 1977 by Kennedy and Knudson [2]. These vascular abnormalities predominate in the lower lung fields. As gravity induces increased blood flow to the lower lung fields hypoxemia is increased when changing from supine to upright position. Mild hypoxemia occurs in approximately one third of all patients with chronic liver disease and often is multifactorial [3,4], because other cardiopulmonary abnormalities (e.g., pleural effusions, ascites) are common in these patients and may coexist with HPS. The special qualities of HPS are platypnea [5], defined as dyspnoe induced by the upright position and relieved by recumbency and orthodeoxia [6], defined as arterial deoxygenation induced by the upright position and relieved by recumbency. Although these phenomena are not pathognomonic for HPS, they strongly suggest this diagnosis in the setting of liver dysfunction [7].

The aim of our open prospective study was to evaluate arterial oxygen saturation (SaO_2_) as a non-invasive screening test for HPS in patients with chronic liver disease or non-cirrhotic portal hypertension. Furthermore, clinical manifestations of HPS were analyzed. Further we wanted to find out, whether it was possible to estimate the shunt volume by this non-invasive procedure.

## Methods

316 in- and outpatients with liver cirrhosis (n = 245), chronic hepatitis (n = 69) or non-cirrhotic portal hypertension (n = 2) were enrolled in a prospective study.

The clinical characteristics of the patients participating in the study are summarized in Table 1. The majority of patients were male (194/316). The mean age was 54.5 years (SD 12.9). The etiology of chronic liver disease was alcohol use in 51%, chronic hepatitis B in 15% and chronic hepatitis C in 22%. Rare aetiologies were hemochromatosis, Wilson's disease, primary biliary cirrhosis (PBC) or autoimmune hepatitis. Two patients had non-cirrhotic portal hypertension due to nodular regenerative hyperplasia (NRH) and portal vein thrombosis of unknown origin, respectively.

The diagnosis of liver cirrhosis was based on clinical, biochemical and ultrasound criteria. In 69/245 patients liver cirrhosis was confirmed histologically. 71 patients had chronic hepatitis or non-cirrhotic portal hypertension without evidence for cirrhosis. The patients with liver cirrhosis were classified according to the Child-Pugh classification [8] as follows: A: 36.3%, B: 42.5% and C: 21.2%.

Measurement of SaO_2 _was performed with a portable pulse oximeter (Onyx, Nonin, USA or Sirecrust 610, Siemens, Germany). In all patients, the measurements were performed at ambient O_2 _partial pressure in supine position. A second reading was taken after 10 minutes in upright position. A pathological test result was defined as: (i) SaO_2 _≤92% in supine position, (ii) a decrease in SaO_2 _of ≥4% after change from supine to the upright position (ΔSaO_2_) [9]. In patients with a pathological test the following additional studies were performed: chest x-ray, pulmonary function tests, transthoracic contrast-enhanced echocardiography (CEE) and perfusion lung scanning.

For CEE, 10 ml of 5.5% oxypolygelatine (Gelifundol^®^, Biotest Pharma, Dreieich, Germany) were injected intravenously. This technique is based on the formation of microbubbles that are normally retained by the pulmonary capillaries and therefore do not appear in the left heart [10]. In the presence of intrapulmonary or intracardiac right-to-left-shunts, however, microbubbles will opacify the left heart. Depending on the time elapsing between contrast medium injection and the appearance of microbubbles in the left heart intracardiac and intrapulmonary shunts can be discriminated. In case of intracardiac right-to-left shunts, microbubbles appear within three heart beats after the appearance in the right chambers. In patients with intrapulmonary shunts contrast medium appears in the left heart after four to six heart beats [11]. To date, there is no satisfactory shunt calculation with the CEE technique [12,13].

Perfusion lung scanning was performed with technetium 99 m (99 mTc)-labeled macroaggregated albumin to directly demonstrate intrapulmonary vascular shunts [14]. Albumin macroaggregates are larger than 20 μm in diameter and are normally trapped in the pulmonary capillaries (diameter 8 to 15 μm). Uptake of radionuclide by kidney, brain etc., therefore, indicates pathological passage through intrapulmonary or intracardiac shunts. The extrapulmonary shunt fraction, assuming that 13% of the cardiac output is delivered to the brain, was calculated using the geometric mean of technetium counts in the brain and lung as described by Abrams and others [12,15]. The shunt fraction was considered positive if the value was >6%.

The diagnosis of HPS was established when the following criteria were fulfilled: arterial deoxygenation (measured by a SaO_2 _≤92 or a ΔSaO_2_of ≥4%), positive CEE and positive perfusion lung scanning using according to the definition of the European Task Force [16].

### Statistical analysis

In our study, quantitative variables are presented as means and standard deviations (SD), qualitative variables as absolute and relative frequencies. Clinical characteristics of HPS patients were compared to those of patients without HPS by a Wilcoxon rank test for quantitative and Fisher's exact test for dichotomous variables. In HPS patients the relationship between CEE and shunt volume was determined by linear regression analysis and estimation of the Pearson correlation coefficient. All tests were two-sided with a p-value of <0.05. Data analysis was performed with the Statistical Analysis System (SAS Institute Inc., Cary, NC, USA). The study protocol was approved by the ethics committee of the Albert-Ludwigs-University of Freiburg. Written informed consent was obtained from all the participants.

## Results

Among the 316 patients seventeen patients (5.4%) had a pathological SaO_2 _according to the criteria described above. These patients underwent CEE analyses (n = 17), perfusion lung scanning (n = 13) and pulmonary function tests (n = 17). On the basis of these analyses 4 patients with HPS were identified. In the 13 non-HPS patients 11 patients had a SaO_2 _< 92 but no remarkable change in upright position and two had a SaO_2 _> 92 combined with a drop of >= 4 in upright position. CEE demonstrated in all four HPS patients the appearance of microbubbles in the left heart 4–6 heart cycles after the appearance in the right heart. In one additional patient microbubbles appeared within three heart beats due to an atrial septum defect. CEE was well tolerated by all patients.

**Figure 1:**
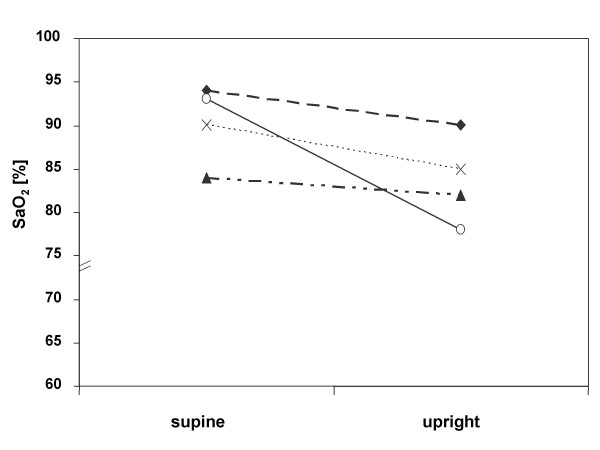
**SaO_2 _in HPS patients**. SaO_2 _in the four HPS patients in supine and after 10 minutes in upright position.

The mean SaO_2 _levels in patients with HPS as compared to patients without HPS were significantly lower in supine (89.7%, SD 5.4 *vs*. 96.0%, SD 2.3, p = 0.0034) and upright position (84.3%, SD 5.0 *vs*. 96.0%, SD 2.4, p = 0.0006, Table 2). Most importantly, not all HPS patients had a pathological SaO_2 _in the supine position but all showed a significant SaO_2 _decrease after changing from supine to upright position (Fig. 1). The mean ΔSaO_2 _in the HPS patients was 5.50 (SD 7) compared to non-HPS patients, who showed no change (p = 0.001). In the 13 patients without HPS the pathologically reduced SaO_2 _was caused by chronic obstructive lung disease (n = 9), large volume ascites, atrial septum defect, the fall of SaO_2 _with normal initial value was seen in a patient with large volume ascites and a patient with portopulmonary hypertension, respectively.

**Figure 2:**
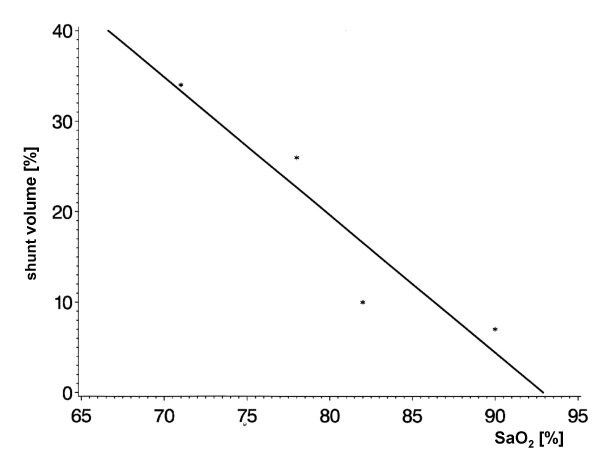
**Correlation of Shunt Volume and SaO_2_**. Correlation between estimated shunt volume (perfusion lung scan) and arterial oxygen saturation (SaO_2_, R = -0.94) in four HPS patients.

**Figure 3:**
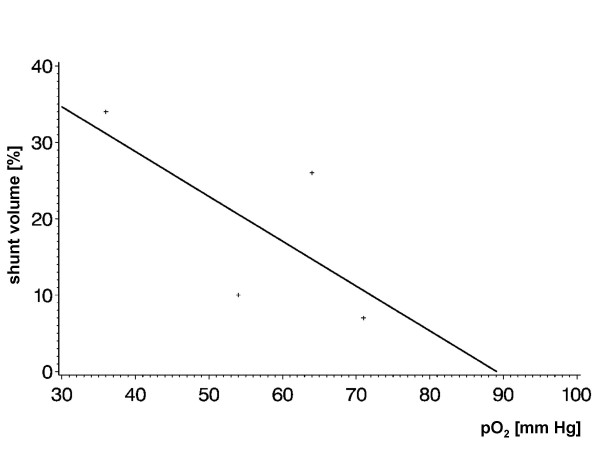
**Correlation of Shunt Volume and arterial pO_2_**. Correlation between estimated shunt volume (perfusion lung scan) and arterial pO_2 _in four HPS patients (R = -0.69).

In 13/17 patients with a pathological SaO_2_, including the 4 HPS patients, a perfusion lung scanning was performed. In all HPS patients extrapulmonary radionuclide uptake was detected. The shunt volume was estimated to be between 7% and 34% (mean 20%) of the cardiac output. The patients without HPS showed no extrapulmonary radionuclide uptake. The shunt volume in the 4 HPS patients correlated very well with the SaO_2 _values (r = -0.94, Fig. 2) and with the pO_2 _values (r = -0.69, Fig. 3).

HPS patients showed pronounced hypoxemia (mean PaO_2 _= 56.2 mm Hg, SD 15.2) in capillary blood gas analyses under ambient O_2 _partial pressure compared to the patients without HPS but a SaO_2 _below 92% (mean PaO_2 _= 78.1 mm Hg, SD 10.7).

In all 17 patients with pathological SaO_2_, pulmonary function tests were performed. CO diffusion capacity (HPS: mean 42%, SD 4.1 *vs*. non-HPS: mean 75.3%, SD 17.7; p = 0.038) and residual volume (2.0 l, SD 0.75 *vs*. 3.3 l, SD 1.2; p = 0.036) were significantly reduced in HPS patients. Vital capacity, pCO_2_, base excess, HCO_3 _and pH were not significantly different in patients with and without HPS, although there was a trend for FEV1 being lower in non-HPS patients.

In HPS patients there was a trend towards higher mean respiration and pulse rate as well as a lower mean systolic and diastolic blood pressure. Cyanosis, dyspnea and digital clubbing were more frequent in HPS patients. The frequency of skin manifestations of chronic liver disease, e.g., spider naevi, palmar erythema, and ascites or hepatic encephalopathy were not different between patients with and without HPS.

Hematologic analyses showed a trend towards higher hemoglobin (14.1 g/dl, SD 2.2 *vs*. 12.5 g/dl, SD 2.2) and hematocrit values (42.5%, SD 6.6 *vs*. 37.5%, SD 12.1) in patients with HPS compared to patients without HPS. Clinical chemistry (bilirubin, albumin, prothrombin time) and Child-Pugh-Score were not different between patients with and without HPS. Chest radiographs showed no abnormalities, such as increased interstitial markings [17], in either group of patients.

## Discussion

Hypoxemia is common in patients with chronic liver disease [17,18]. A rare cause is HPS that may cause dyspnea, platypnea, and orthodeoxia and poor prognosis [19]. Because other abnormalities, (e.g., hydrothorax, ascites) may coexist in HPS patients and contribute to respiratory insufficiency, measurement of a lowered paO_2 _or SaO_2 _alone is not sufficient to make the diagnosis of HPS. The diagnostic criteria of HPS include liver disease, hypoxemia with a paO_2_<70 mmHg and evidence for intrapulmonary vascular dilation. This study was conducted to evaluate pulse oximetry as a non-invasive screening for HPS in patients with chronic liver disease. Therefore, arterial blood gas analyses were only obtained from patients with a SaO_2 _below the threshold value of 92% or a decrease in SaO_2 _of ≥4% after change from supine to the upright position (ΔSaO_2_). In liver transplant candidates a threshold level of SaO_2 _of 94% detected all subjects with an arterial pO_2 _<60 mm Hg [20]. A patient with a SaO_2 _>92% and no significant decrease in this value in the upright position is unlikely to have a paO_2 _<70 mm Hg and pulmonary shunts without these being detected by our screening. There may be a small subgroup of patients with positive contrast echocardiograms and essentially normal or slightly changed oxygenation due to form fruste or subclinical HPS. We cannot determine how many patients we missed to detect due to a lack of a position change in SaO_2_. The incidence and clinical significance of these forms of HPS is not clear. However, patients with clinically apparent HPS have a significant mortality and have to be identified, because HPS is an indication and not a contraindication for liver transplantation [21-23]. Our intention was not to determine the true prevalence of HPS including minor forms but to detect clinically relevant cases by a simple screening algorithm.

In our study of 316 patients almost 80% had liver cirrhosis; the remaining patients had chronic hepatitis or non-cirrhotic portal hypertension. The majority of patients had compensated liver function. Only 17 patients (5.4%) had a pathological SaO_2 _or a decrease in SaO_2 _of ≥4%. This is a relatively small percentage compared to other study populations, such as liver transplant candidates [12]. Four patients (1.3%) in our prospective study met the diagnostic criteria for HPS. 11 patients without HPS had a SaO_2 _≤92 and two a ΔSaO_2 _of ≥4%.

Data regarding the incidence of HPS are limited and estimations of prevalence vary as different definitions for HPS are used. Based on the prospective design of our study, the 1.3% prevalence of significant HPS appears realistic in patients with chronic liver disease.

HPS patients in our study were younger than patients without HPS. Similarly, Rydell and Hoffbauer in 1956 described a 17-year old patient with liver cirrhosis and dilated pulmonary vessels and arteriovenous fistulas, found at autopsy [24]. At the same time, Hales described some very young patients with similar pulmonary findings and liver disease [25]. Even in childhood HPS may occur [26].

Two of our 4 HPS patients had liver cirrhosis due to chronic viral hepatitis. One of these patients also had a hepatocellular carcinoma. The two other HPS patients had portal hypertension due to NRH and portal vein thrombosis of unknown origin, respectively. Both acute [27] and chronic [28,29] liver diseases have been associated with HPS. Most commonly, HPS appears in patients with chronic liver diseases progressing to liver cirrhosis, especially in cryptogenic cirrhosis, alcoholic cirrhosis, PBC and chronic viral hepatitis B or C. However, the occurrence of HPS in patients with noncirrhotic portal hypertension [28,30], as in 2 of our HPS patients, suggests that cirrhosis is not a prerequisite for the development of HPS.

In our patients there was no correlation between the occurrence of HPS and liver function tests (bilirubin, albumin and prothrombin time), similar to previous findings [28,31]. Different from other studies [32,33] we found no association between spider naevi or other skin manifestations of liver disease and HPS. The most impressive physical findings in HPS patients were cyanosis, dyspnea and digital clubbing, which were more frequent in HPS patients than in non-HPS patients. The pathogenesis of digital clubbing is not completely understood. One hypothesis is based on the fact that megakaryocytes and platelet aggregates are normally retained by the lung capillaries. In patients with a right-to-left-shunts megakaryocytes reach the digital capillaries [34,35], there releasing platelet derived growth factor (PDGF). PDGF is known to cause increased capillary permeability and proliferation of fibroblasts that may result in clubbing [36].

Since hypoxemia and orthodeoxia are not pathognomic for HPS, CEE is necessary to discriminate between intrapulmonary or intracardiac right-to-left shunts. All patients with pathological SaO_2 _or elevated ĆSaO_2 _underwent CEE in our study. In 5 patients microbubbles appeared in the left heart. In one patient this was caused by an atrial septum defect. In the remaining four patients HPS was diagnosed, because microbubbles appeared already after 4–6 heart beats in the left heart.

^99m^Tc-labeled macroaggregated albumin scanning is another technique to detect and to quantify intrapulmonary vascular shunts [12,15]. In all 4 HPS patients extrapulmonary radionuclide uptake confirmed the existence of intrapulmonary shunts. The estimated shunt volume was between 7% and 34% (mean 20%) of the cardiac output and correlated well with the SaO_2 _(r = -0.94) and the arterial pO_2 _(r = -0.69). Abrams and coworkers described a similar correlation between arterial pO_2 _and shunt volume (r = -0.73) however, SaO_2 _was not determined [15].

Patients with end-stage liver disease may have various pulmonary abnormalities, including restrictive and obstructive lung disease. HPS patients tend to have a decreased CO diffusion capacity with normal pulmonary capacity and expiratory flow rates [17,37]. These findings were confirmed in our study.

## Conclusion

Combined SaO_2 _determination in supine and upright position using a pulse oximeter is a simple test to identify HPS in patients with chronic liver disease or noncirrhotic portal hypertension. In our prospective clinical study only in 4 out of 316 patients (1.3%) with chronic liver disease had HPS. Shunt volumes can be quantified by lung perfusion scanning and correlate better with the values of SaO_2 _than pO_2_. This correlation may allow an estimation of the severity of the shunt volume without performing lung perfusion scanning. In clinical practice determination of arterial oxygen saturation with a pulse oximeter can be used as a simple screening test and may help to identify more HPS patients with the aim to study the pathogenesis and to develop therapeutic strategies.

## Competing interests

The author(s) declare that they have no competing interests.

## Authors' contributions

Peter Deibert is the corresponding author of the study. Peter Deibert, Hans-Peter Allgaier and Stefanie Loesch took part in designing the study, performed the data analyses and drafted the manuscript. Claudia Müller performed data acquisition in a considerable number of patients and helped to write the manuscript. Manfred Olschewski supported data analyses. Hinrich Hamm, Klaus-Peter Maier and Hubert Erich Blum supervised the study and gave critical comments to the manuscript. All authors have read and approved the final version of the manuscript.

## Pre-publication history

The pre-publication history for this paper can be accessed here:



## Figures and Tables

**Table 1:** Characteristics of the 316 Study Patients

Variable	
Mean Age* (yr)	54.5 (12.9)
Sex (M/F)	194/122
Etiology of liver disease [%]	
Alcohol	51.2
Hepatitis B virus	15.2
Hepatitis C virus	22.2
Hemochromatosis	1.9
Wilson's diesease	0.6
PBC	1.9
Autoimmune hepatitis	2.8
Unknown	4.2
Manifestation of liver disease [%]	
Chronic hepatitis (n = 69)	21.8
Liver cirrhosis (n = 245)	77.5
Non-cirrhotic portal hypertension (n = 2)	0.6
Liver cirrhosis classification [%]	
Child-Pugh-Stage A	36.3
B	42.5
C	21.2

**Table 2:** Characteristics of the 4 Patients with HPS

Age (yr)	Gender	Disease etiology	SaO_2 _(%) supine	SaO_2 _(%) upright	pO_2 _(mmHg) upright	Shunt volume (%)
62	†	HCV-cirrhosis	94	90	71	7
28	♂	Portal vein thrombosis	84	82	54	10
27	♂	Chronic EBV-infection	93	78	64	28
31	♂	Nodular regenerative hyperplasia	90	71	36	34
